# Different Regional Gray Matter Loss in Recent Onset PTSD and Non PTSD after a Single Prolonged Trauma Exposure

**DOI:** 10.1371/journal.pone.0048298

**Published:** 2012-11-14

**Authors:** Yunchun Chen, Kuang Fu, Chen Feng, Lihua Tang, Jian Zhang, Yi Huan, Jinli Cui, Yunfeng Mu, Shun Qi, Lize Xiong, Cheng Ma, Huaihai Wang, Qingrong Tan, Hong Yin

**Affiliations:** 1 Department of Psychiatry, Xijing Hospital, Fourth Military Medical University, Xi’an, Shaanxi, China; 2 Department of Magnetic Resonance Imaging, The Second Affiliated Hospital, Harbin Medical University, Harbin, Heilongjiang, China; 3 Department of Radiology, Xijing Hospital, Fourth Military Medical University, Xi’an, Shaanxi, China; 4 Department of Radiology, PLA General Hospital, Beijing, China; 5 Department of Respiratory Medicine, Xijing Hospital, Fourth Military Medical University, Xi’an, Shaanxi, China; 6 Department of Anesthesiology, Xijing Hospital, Fourth Military Medical University, Xi’an, Shaanxi, China; University of Massachusetts Medical School, United States of America

## Abstract

**Objective:**

Gray matter loss in the limbic structures was found in recent onset post traumatic stress disorder (PTSD) patients. In the present study, we measured regional gray matter volume in trauma survivors to verify the hypothesis that stress may cause different regional gray matter loss in trauma survivors with and without recent onset PTSD.

**Method:**

High resolution T1-weighted magnetic resonance imaging (MRI) were obtained from coal mine flood disaster survivors with (n = 10) and without (n = 10) recent onset PTSD and 20 no trauma exposed normal controls. The voxel-based morphometry (VBM) method was used to measure the regional gray matter volume in three groups, the correlations of PTSD symptom severities with the gray matter volume in trauma survivors were also analyzed by multiple regression.

**Results:**

Compared with normal controls, recent onset PTSD patients had smaller gray matter volume in left dorsal anterior cingulate cortex (ACC), and non PTSD subjects had smaller gray matter volume in the right pulvinar and left pallidum. The gray matter volume of the trauma survivors correlated negatively with CAPS scores in the right frontal lobe, left anterior and middle cingulate cortex, bilateral cuneus cortex, right middle occipital lobe, while in the recent onset PTSD, the gray matter volume correlated negatively with CAPS scores in bilateral superior medial frontal lobe and right ACC.

**Conclusion:**

The present study identified gray matter loss in different regions in recent onset PTSD and non PTSD after a single prolonged trauma exposure. The gray matter volume of left dorsal ACC associated with the development of PTSD, while the gray matter volume of right pulvinar and left pallidum associated with the response to the severe stress. The atrophy of the frontal and limbic cortices predicts the symptom severities of the PTSD.

## Introduction

Gray matter volume reductions in limbic structures and hippocampus have been found in trauma survivors with recent onset post traumatic stress disorder (PTSD) [1]–[4]. These results indicated that the gray matter of limbic region and hippocampus involve in the development of PTSD. However, the longitudinal study about recent onset PTSD did not find hippocampus volume change [5], suggesting hippocampus volume reduction may not be a necessary condition for recent onset PTSD.

The stress plays an important role in the onset of PTSD. In previous studies, the population studied consisted of PTSD and trauma exposed non-PTSD subjects [2], [4], or PTSD and normal controls without any trauma exposure [1], [3]. However, no study included PTSD, non-PTSD and normal controls together. Therefore, it is difficult to answer the question that PTSD is associated with the results of different scale of damage to the same brain structure or damage of different brain structures. The functional MRI (fMRI) study demonstrated that the stress can not only cause hyper regional activity in frontolimbic and striatal areas, but also can cause decreased functional connectivity between limbic and striatal networks soon after the trauma in trauma survivors [6]. The stress also can cause limbic structure atrophy similar as PTSD in trauma survivors without PTSD [7]–[8]. These findings suggested that stress can cause the similar functional and structural abnormalities as PTSD in trauma survivors without PTSD. So it is better to take PTSD and non PTSD with similar trauma experience and demographic characteristics, and no trauma exposed normal controls as study subjects, to investigate the effects of stress on the gray matter volume.

A severe coal mine-flood disaster occurred in central China on July 29, 2007, 69 male miners were trapped in a nearly 1400 m underground coal pit, all of them were fortunately rescued after 72 h of the ordeal in the darkness [9]. A part of the coal mine-flood survivors developed to PTSD syndromes which expressed as interpersonal symptoms, anxiety, paranoid ideation, and psychoticism. Those trauma survivors had considerable homogeneity in demographic variables and traumatic type, intensity, and duration of exposure, and avoided most comorbidity factors, that offered a distinct advantage in examining psychological consequences of PTSD and stress on brain structure.

Voxel-based morphometry (VBM) method was used to measure the gray matter volume in coal mine flood disaster survivors with and without recent onset PTSD, compared with a group of age and gender matched no trauma exposed normal controls. Based on the results of previous studies, we hypothesize that gray matter volume may loss in different regions in recent onset PTSD patients and non PTSD subjects relative to normal controls. The severity of PTSD symptoms may correlate with the brain structure in the trauma survivors including the recent onset PTSD and non PTSD.

## Materials and Methods

### Subjects

A trauma survivor group (n = 20) and a normal control group (n = 20) were enrolled in the present study. All the trauma survivor group members were from the survivors of a coal mine flood disaster that occurred in July 29, 2007 in the Henan province of China. As a program of psycho-aid organized by the government, the clinical evaluation and diagnosis of the members were carried by a psychiatric team from Xijing Hospital who were trained to expertly use the DSM-IV [10] and the Structured Clinical Interview for DSM-IV (SCID) [11] about 3 months after the disaster. And the initial diagnosis was made by the two psychiatrists, and confirmed by a senior psychiatrist on the basis of a direct interview 6 months after trauma, 17 of the traumatic members were diagnosed as recent onset PTSD, 10 of them agreed to attend MR study, there were no significant differences in symptom severity and demographic characteristics between 10 PTSD patients and the other PTSD patients who did not join the MRI study, while 10 of trauma survivors without PTSD agreed to attend the MRI study, there were no significant differences in symptom severity and demographic characteristics between 10 non PTSD subjects and the other non PTSD subjects who did not join the MRI study. The severity of their symptoms was assessed with the Clinician-Administered PTSD Scale (CAPS), and Symptom Check List 90 (SCL90). The normal control group members came from no trauma exposed health volunteers, none of the subjects met diagnostic criteria for major depression, schizophrenia or bipolar disorder. The identified PTSD patients had never received psychiatric treatment for their condition. Moreover, none of the subjects had a history of treatment with psychotropic drugs or of substance (alcohol, smoking and drug) abuse. All study subjects did not have a history of prior trauma exposure, and all of them were right handed male, free of metallic implants, neurological disorder, and major medical conditions. High resolution MRI examinations were carried on all the members of the trauma survivor group and control group. The duration times between the traumatic event and the MRI scans ranged from 187 to 190 days. All participants gave voluntary, written, informed consent before entering the study, and the study protocol was approved by the Medical Ethical Committee of Xijing Hospital of the Fourth Military Medical University.

### Magnetic Resonance Imaging Acquisition

All MRI scans were performed on a 3.0 T MR scanner (MAGNETOM Trio, Siemens AG, Erlangen, Germany) equipped in the Department of Radiology, Xijing Hospital. A high-resolution three dimensional magnetization prepared rapid acquisition gradient echo (MPRAGE) T1-weighted sequence was used to acquire MR images over the whole-brain (176 sagittal slices). Other MR imaging parameters applied in this study were TR = 1900 ms, TE = 2.26 ms, TI = 900 ms, flip angle = 9°, acquisition matrix = 256×256, field of view = 220 mm and 1.00 mm slice thickness with no inter-slice gap.

### MRI Analysis

Image analysis was performed on a computer workstation with MATLAB 7.11.01(MathWorks, Natick, USA) and SPM8 software (Wellcome Department of Cognitive Neurology, London) with methods similar to those described previously [12].

In the preprocessing step of VBM, DARTEL was used to improve inter-subject registration of structural images. The following processing steps were carried out: (1) the artifacts of raw data for each subject were inspected and image origin was set at the anterior commissure; (2) structural images of the 20 normal subjects were used to make DARTEL templates. (3) Structural images of each subject were applied to unified segmentation and initial import, and the imported data were warped to existing DARTEL templates, and flow fields files were generated; (4) by combining the flow fields and imported data, the Jacobian scaled warped tissue images were generated through Jacobian modulation [13] in order to preserve the initial volumes;(5)the warped data were smoothed with a 12 mm FWHM for statistical analysis. The modulated gray matter volume, white matter volume and cerebral spinal fluid (CSF) volume were summed to get total intracranial volume (TIV).

### Statistical Analyses

Group differences in demographic and clinical variables were examined with analysis of variance (ANOVA) in SPSS11.0 (SPSS, Inc, Chiago.IL), considering there is a significant difference in age between PTSD and non-PTSD groups, the gray matter volume, white matter volume and CSF volume were analyzed with analysis of covariance (ANCOVA) with age as covariate. Group comparisons for gray matter volume were performed in SPM8 with ANCOVA, with diagnosis as a fixed factor, education years, age and TIV as covariates. Two sides of contrasts were examined: normal controls>comparison subjects and normal controls<comparison subjects. The statistical significance threshold was set at *p*<0.05 corrected for multiple comparison using the Family Wise Error (FWE), with a minimum of 50 contiguous voxels required [14].

To test the hypotheses about regionally specific covariate effects, in the trauma survivor group, the correlation study between the gray matter volume and the CAPS and SCL90 were performed using multiple regression in the basic model of the SPM8, using CAPS, SCL90 as predict factor, age and TIV were treated as confounding covariates. Two sides of correlations (positive or negative) were examined, and significance level was set to *p*<0.05 (corrected at cluster level for multiple comparison), with a minimum of 100 contiguous voxels required.

## Results

### Demographic

The normal controls and the trauma survivors did not differ in age, but differed in years of education (t = 10.07; df = 38, P = 0.001). In the trauma survivors, the PTSD and non PTSD differed in age (t = 2.318; df = 18, P = 0.032), but did not differ in years of education. All trauma survivors came from the same community and did not differ significantly in socioeconomic status. As expected, the PTSD patients had higher CAPS scores than the non PTSD subjects [t = 5.895; P = 0.000], and there was significant difference in SCL90 between the PTSD and non PTSD (t = −2.43, p = 0.026), there was also significant difference between PTSD and non PTSD in re-experience (t = −3.83, df = 18, p = 0.001), avoidance (t = −4.21, p = 0.001), arousal (t = −2.97, p = 0.008)and intrusion (t = −4.76, p = 0.0001) (Table 1). The gray matter volume of PTSD and non PTSD did not show significant difference compared with normal controls.

**Table 1:** Sociodemographic and Volumetric Characteristic of the Sample.

Variable	trauma survivors				
	PTSD (n = 10) Mean±SD	non PTSD (n = 10) Mean±SD	p	normal control Mean±SD	P
Age,yrs	40.8±6.8	34.3±5.6	0.032	37.6±7.0	0.64
Length of education,yrs	7.2±1.7	8.2±1.5	0.19	14.1±2.6	0.001
Caps	78.7±17.3	34.4±18.6	0		
SCL90	25.02±10.8	15.8±5.02	0.026		
Reexp	23.6±4.14	15.4±5.46	0.001		
Avoid	20.7±4.55	12.9±3.69	0.001		
Arous	19.4±3.966	13.3±5.9	0.008		
impair	7.8±1.67	3.9±1.96	0.001		
Gray matter volume (cm^3^)	634.34±49.01^a^	661.36±66.72^b^	0.93	687.71±57.96	0.03
White matter volume (cm^3^)	577.75±55.98	588.86±63.77	0.76	572.06±51.12	0.48
CSF volume (cm^3^)	200.91±13.49	201.35±22.93	0.96	240.94±23.59	0.004

a There is no significant difference in gray matter volume between PTSD group and normal controls, p = 0.07.

b There is no significant difference in gray matter volume between non PTSD group and normal controls, p = 0.08.

PTSD: Post Traumatic Stress Disorder.

### Recent Onset PTSD vs Normal Controls

**Figure 1:**
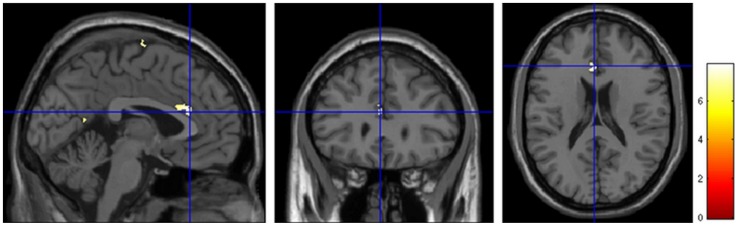
Regional gray matter volume reduction in recent onset PTSD compared with normal controls. The significant reduction regions in trauma survivors with recent onset PTSD (n = 10) compared with normal controls (n = 20) are rendered onto the standard T1 template of Montreal Neurological Institute. The trauma survivors with recent onset PTSD had a significantly decreased gray matter volume in the left ACC (*p*<0.05, FWE corrected, with *k*>50 voxels). ACC: anterior cingulate cortex.

The patients with recent onset PTSD had significantly smaller gray matter volume in the left anterior cingulate cortex (ACC) compared with the normal controls (Figure 1, Table 2).

**Table 2:** Gray matter volume differences between recent onset PTSD, non PTSD and normal Controls.

Group	Brain regions	MNI coordinates			Clustersize	Zscore	p value
		X	Y	Z			
Normal control (n = 20) > Recent onset PTSD (n = 10)							
	Left rostral anterior cingulate cortex	−6	26	20	207	5.25	0.004
Normal control (n = 20) > non PTSD (n = 10)							
	left pallidum	−27	−15	−3	131	4.92	0.013
	right pulvinar	21	−24	26	813	5.92	0.0001

Corrected with Family Wise Error (FWE), p<0.05.

MNI: Montreal Neurological Institute system.

PTSD: Post Traumatic Stress Disorder.

### Non PTSD vs Normal Controls

**Figure 2:**
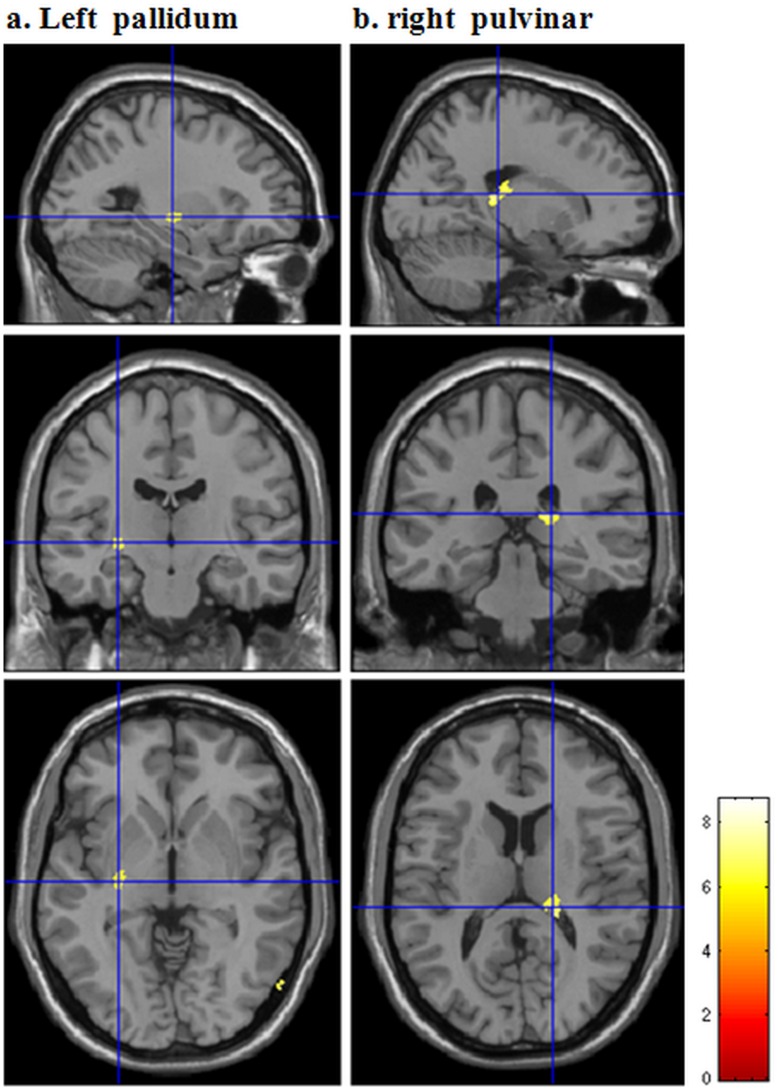
Regional gray matter volume changes in trauma survivors without PTSD compared with normal controls. The significant reduction region in trauma survivors without PTSD compared with normal controls (n = 20) are rendered onto the standard T1 template of Montreal Neurological Institute. Compared with normal controls, trauma survivors without PTSD (n = 10) had significantly decreased gray matter volume in left pallidum and right pulvinar. (*p*<0.05, FWE corrected, with *k*>50 voxels).

The non PTSD subjects showed significantly smaller gray matter volume in left pallidum and pulvinar of the right thalamus compared with the normal controls (Figure 2, Table 2).

### PTSD vs Non PTSD

No significant difference was identified in gray matter volume between PTSD and non PTSD group.

### Correlations

**Figure 3:**
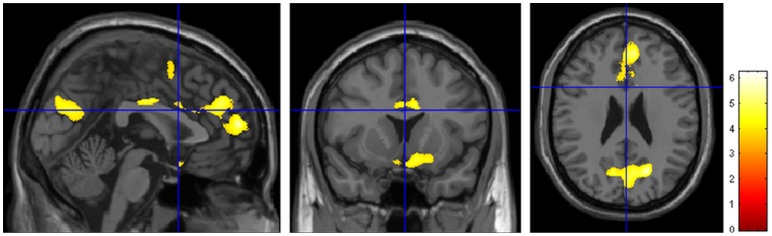
Regional gray matter volume correlations with CAPS score in trauma survivors. In the trauma survivors (n = 20), the gray matter volume significantly and negatively correlated with the CAPS scores in the bilateral superior medial frontal lobe, left anterior and middle cingulate cortex, bilateral cuneus cortex, right middle occipital lobe. (*p*<0.05, corrected at cluster level, with *k*>100 voxels).

In all trauma survivors including both non PTSD and PTSD, the gray matter volume correlated significantly and negatively with the CAPS scores in the bilateral superior medial frontal lobe, left anterior and middle cingulate cortex, bilateral cuneus cortex, right middle occipital lobe (Figure 3, Table 3).

**Table 3:** Correlations of Gray matter volume with CAPS score in trauma survivors, recent onset PTSD and non PTSD.

	Brain regions	MNI coordinates			Clustersize	Zscore	
		X	Y	Z			p value
trauma survivors							
	right cuneus	18	−67	28	5261	4.27	0.0001
	right middle occipital lobe	32	−87	5	152	3.74	0.0001
	right frontal lobe	4	44	27	1150	3.97	0.0001
	left cunes	−13	−69	26	1025	3.91	0.0001
	left anterior and middle cingulate cortex	−9	−6	32	2010	3.92	0.0001
PTSD group							
	right superior medial frontal lobe	5	42	30	1361	4.83	0.0001
	right anterior cingulate cortex	12	46	2	495	4.83	0.003
	left superior medial frontal lobe	−6	61	12	891	4.1	0.004
non PTSD group	no						

corrected at cluster level, p<0.05.

MNI:Montreal Neurological Institute system.

PTSD: Post Traumatic Stress Disorder.

**Figure 4:**
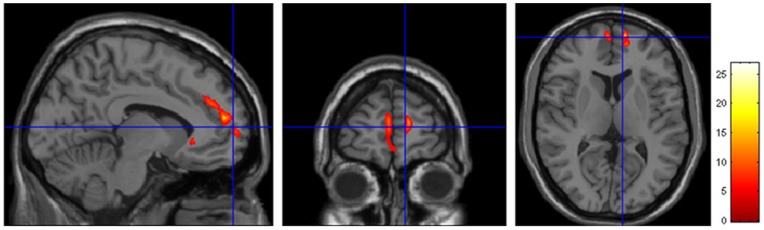
Regional gray matter volume correlations with CAPS score in trauma survivors with recent onset PTSD. In trauma survivors with recent onset PTSD (n = 10), the gray matter volume significantly and negatively correlated with the CAPS scores in the bilateral superior medial frontal lobe and right anterior cingulate cortex (p<0.05, corrected at cluster level, with k>100 voxels).

In recent onset PTSD patients, the gray matter volume correlated significantly and negatively with the CAPS scores in the bilateral superior medial frontal lobe and right ACC (Figure 4, Table 3). While in the non PTSD subjects, no significant correlations between the gray matter volume and CAPS scores were detected.

There was no significant correlation between SCL90 and gray matter volume in trauma survivors, PTSD group or non PTSD group.

## Discussion

There are three major findings in the present study. First, atrophy in the left dorsal ACC of trauma survivors with recent onset PTSD was identified, compared with normal controls. Second, atrophy in left putamen and right pulvinar of trauma survivors without PTSD was identified compared with normal controls. Third, the gray matter volume of trauma survivors correlated negatively and significantly with CAPS scores in bilateral superior medial frontal cortex, anterior and middle cingulate cortex, bilateral cuneus cortex, right occipital cortex. Furthermore, in recent onset PTSD patients, the gray matter volume correlated negatively with the CAPS scores in bilateral superior medial frontal cortex and right ACC. These results implicated that the bilateral frontal lobe and right ACC associate with PTSD symptom severity. For the unique characteristic of the trauma event and homogeneous demographic characteristic of trauma survivors, the present study results implicated that gray matter loss in specific region at 6 months after a single prolonged trauma may associate with PTSD onset and stress response.

In the present study, we found a significantly decreased gray matter volume in left dorsal ACC in recent onset PTSD patients compared with normal controls, which indicated that the left dorsal ACC may specifically associate with developing of recent onset PTSD at early stage. This result is partially consistent with previous studies about recent onset PTSD [2]–[3] and chronic PTSD [15]–[16]. In previous studies, besides the findings of gray matter volume loss, increased fractional anisotropy was identified in subjacent white matter of the left dorsal ACC in PTSD, represented the altered integrity of adjacent white matter [17]. Acute stress also can cause hyperactivity of prefrontal-limbic and striatal systems [18], and there is attenuated functional connectivity involving limbic-striatal areas as early as 25 days after trauma [6], indicates the stress can cause both functional and structure changes in left prefrontal cortex and the limbic regions. The ACC, the amygdala and the hippocampus [19] are important structures in the processes involved in normal fear extinction [20]–[21] and regulation of emotion [22], these regions showed dysfunction in patients with chronic PTSD [23]. There are also studies showed stronger functional connectivity between the ACC and limbic cortices,and that may contribute to emotion processing in PTSD [24]–[26].Successful extinction of learned fear depends upon activity of amygdala N-methyl-D-aspartate (NMDA) receptors [20], while decreased ACC activity following extinction appears to be associated with persistence of fear responses [21]. Thus, the ACC may act to facilitate fear extinction by modulating the amygdala and dysfunction of ACC may result in failure to extinguish learned fear [27]. A number of functional imaging studies results suggested that excessive amygdala activity and reduced ACC activity are present in PTSD [28]. Functional imaging studies also identified greater activation of the amygdala, anterior paralimbic structures, and failure of activation of ACC in response to trauma related stimuli in individuals with PTSD [5], [29].

Ventromedial prefrontal cortex (vmPFC) is implicated in fear extinction in animal [30]–[32] and human [18], [33]. Furthermore, cortical thickness of the vmPFC is positively correlated with extinction of learning in humans [34].These findings are consistent with hypothetical models that PTSD is maintained by elevated amygdala reactivity that is inadequately regulated by vmPFC [28]. This model is also supported by evidence that PTSD patients display reduced activation of the rostral anterior cingulate cortex (rACC) during fear processing [35]. A range of studies have investigated the volume of ACC in PTSD patients [36]–[37], and meta-analysis of these studies indicates that PTSD patients have smaller ACC volume than trauma exposed people without PTSD [38]. The present study results indicate that left ACC may involve in the PTSD at early stage.

Different from previous studies [1]–[2], the present study did not find atrophy in hippocampus and parahippocampal gyrus or insular cortex, which may be due to the following reasons, first, in previous studies about recent onset PTSD, the trauma exposure time is short or different in individuals [2]–[3], and trauma types are different [1]. Although there is no evidence showing that different trauma exposure time and trauma type associate with specific brain structure change in PTSD, the previous reports of brain structural damages are inconsistent in regards to recent onset or chronic PTSD [4]. For the trauma severity associates with PTSD symptom severity [39], the strength of traumatic exposure is significantly related to PTSD severity [40]–[43], the accumulated traumatic experience negatively correlated with limbic structure gray matter density [44], therefore, it is possible that different trauma type and exposure time may have different effects on brain structure in recent onset PTSD and trauma survivors without PTSD. Second, in previous studies about recent onset PTSD, the analyses were performed only between PTSD and non PTSD [2], [4] or between PTSD and normal controls [1], [3], no study investigated brain structure differences between PTSD, non PTSD and normal controls. It is known that the trauma exposure not only associated with PTSD, but also associated with comorbidity such as depression. Stress can cause brain structure changes not only in PTSD patients, but also in non PTSD subjects [7]–[8]. According to fear response neurocircuit, there is possibility that brain structure change in recent onset PTSD and non PTSD may overlap after a period of time, and that was found in trauma victims [7]. However, the early brain structure changes in PTSD and non PTSD were not well explored. In the present study, we found that single prolonged trauma exposure can cause different regional gray matter loss in recent onset PTSD and non PTSD as compared to normal controls. This finding verified our hypothesis that PTSD patients and non PTSD subjects have different region of gray matter loss at early stage after trauma. This is the first report that single prolonged trauma exposure causes different region’s gray matter loss in PTSD and non PTSD at 6 months after trauma.

The dorsomedial thalamus receives input from the amygdala and is a key structure for visual input into the prefrontal cortex [45]–[46]. It belongs to the circuitry activated by fear-inducing stimuli [47]. The dorsomedial thalamus is also involved in fear processing [48]. The dorsal pallidum is related to the operative control of behavior [49], and there was a neuron degeneration of this region in depressed rats [50]. The deep brain stimulation (DBS) in the thalamus and globus pallidus region can cause mood, cognitive, and behavioral changes like a PTSD syndrome [51], demonstrating the role of thalamus in fear processing. Thus, in non PTSD subjects of present study, the clinical symptom may partially associate with the atrophy in the left pallidum and right pulvinar. The similar structural changes were also found in chronic PTSD patients [52].

As a protective and adaptive mechanism, the proper function of fear conditioning depends on the integration of content and context information [53]. The proper function of the limbic system, especially the amygdala, depend on control from bilateral supp-motor area, frontal-sup-medial cortex, anterior and middle cingulate cortex to discriminate between the threat and no threat stimuli [54], [55]. Therefore, in traumatic fear conditioning, the limbic system’s activation might lead more load than normal controls to superior medial frontal cortex, anterior and middle cingulate cortex, and finally result gray matter loss in above regions. In present study, we found that in the bilateral cuneus cortex, right frontal cortex, left anterior and middle cingulate cortex, and right middle occipital lobe, the gray matter volume of trauma survivors negatively correlated with their CAPS scores, and the PTSD subgroup had smaller region of gray matter than the total trauma survivor group that negatively correlated to the CAPS scores. This may be an evidence to support the mechanism mentioned above. In the PTSD subgroup, there was only bilateral superior medial frontal cortex and right ACC responding to the modulation of vigilant status, while in the whole trauma survivor group there was a much larger region involved in the reflection, suggesting that the PTSD may be regional specific. Another potential reason for the results is that the PTSD group has a smaller number of subjects than the trauma survivor group, but the concrete role of above regions in recent onset PTSD is still uncertain.

Compared with the normal control group, there is no significant difference in gray matter volume between the PTSD and non PTSD in present study. That is because in previous study [4], we did find the gray matter loss in the left hippocampus and parahippocampal gyrus and bilateral calcarine cortex in PTSD relative to non PTSD, in that study, the correction for multiple comparison is at cluster level, but in the present study, the correction for multiple comparison at FWE level, that is more strict than previous study.

The present study also has a number of potential limitations. First, we acknowledge that VBM, though robust for larger samples, is not superior to expert manual segmentation, and the transformations required for VBM may induce artificial volumetric differences (such as mis coregistration) and the VBM method may not detect the gray matter volume change in amygdala and subfield of hippocampus [56]. Second, our sample of PTSD is small, and this makes some of statistical conclusions uncertain. Third, we did not get enough longitudinal imaging data of trauma survivors, so we could not identify whether the current results are preexisting or acquired damage, and we could not analyze the switch-point of the gray matter volume changes and their relationship with development of PTSD. Finally, in the present study, the normal controls were more educated than trauma survivors, and were not from same community, these difference may have effect on the present study results, so it is better to take coal miner form same community as normal controls.

Taken together, in trauma survivors of a unique prolonged trauma event, we found recent onset PTSD and non PTSD had different regions of gray matter loss at 6 months after the trauma, indicated that the gray matter loss in left ACC may associate with the PTSD onset, while gray matter loss in the pulvinar and pallidum may involve in the stress response. The atrophy of frontal-limbic cortex in recent onset PTSD may predicate PTSD symptom severities.
